# De Novo T790M Mutation in an L858R Epidermal Growth Factor Receptor Mutant-Associated Lung Adenocarcinoma

**DOI:** 10.3390/cancers12103074

**Published:** 2020-10-21

**Authors:** Takumi Fujiwara, Tetsu Kobayashi, Taro Yasuma, Corina N. D’Alessandro-Gabazza, Masaaki Toda, Hajime Fujimoto, Kentaro Fujiwara, Atsuro Takeshita, Kota Nishihama, Tomohito Okano, Valeria Fridman D’Alessandro, Yoshiyuki Takei, Osamu Hataji, Esteban C Gabazza

**Affiliations:** 1 Department of Pulmonary and Critical Care Medicine, Graduate School of Medicine, Mie University, Mie, Edobashi 2-174, Tsu, Mie 514-8507, Japan; t-fujiwara@clin.medic.mie-u.ac.jp (T.F.); ktetsu@clin.medic.mie-u.ac.jp (T.K.); fjmt1974@clin.medic.mie-u.ac.jp (H.F.); okatomojin525@clin.medic.mie-u.ac.jp (T.O.); ytakei@clin.medic.mie-u.ac.jp (Y.T.); 2 Department of Immunology, Faculty and Graduate School of Medicine, Mie University, Edobashi 2-174, Tsu, Mie 514-8507, Japan; t-yasuma0630@clin.medic.mie-u.ac.jp (T.Y.); dalessac@clin.medic.mie-u.ac.jp (C.N.D.-G.); t-masa@doc.medic.mie-u.ac.jp (M.T.); atsurolennon@clin.medic.mie-u.ac.jp (A.T.); immunol@doc.medic.mie-u.ac.jp (V.F.D.); 3 Respiratory Center, Matsusaka Municipal Hospital, Tonomachi1550, Matsusaka, Mie 515–8544, Japan; mchfujiwara@city-hosp.matsusaka.mie.jp (K.F.); mch1031@city-hosp.matsusaka.mie.jp (O.H.); 4 Department of Diabetes, Metabolism, and Endocrinology, Mie University Graduate School of Medicine, Edobashi 2–174, Tsu, Mie 514-8507, Japan; k-nishihama@clin.medic.mie-u.ac.jp

**Keywords:** epidermal growth factor receptor, driver mutations, lung adenocarcinoma, drug resistance, tyrosine kinase inhibitor

## Abstract

**Simple Summary:**

Lung adenocarcinomas caused by (L858R) somatic mutation in the epidermal growth factor receptor (EGFR) show good therapeutic response to tyrosine kinase inhibitors. These tumors develop resistance to therapy mainly after acquired T790M mutation. However, whether the T790M mutation occurred before or after therapy is unknown. To demonstrate this, we developed mice with tetracycline-inducible lung-specific expression of the full-length genomic DNA of the human epidermal growth factor receptor containing an L858R mutation or both L858R and T790M mutations and evaluated de novo T790M mutation in untreated transgenic mice carrying a single L858R EGFR mutation. The results showed that lung tumors spontaneously acquire T790M mutation without any drug-related selective pressure.

**Abstract:**

*Background:* Lung cancer is the leading cause of mortality for cancer worldwide. A point mutation in exon 21 of the epidermal growth factor receptor resulting in the substitution of arginine for leucine at position 858 (L858R) is a frequent cause of lung adenocarcinoma. Tyrosine kinase inhibitors are effective for treating patients with lung cancer associated with mutant epidermal growth factor receptors but most tumors become resistant shortly after treatment. The substitution of methionine for threonine at position 790 (T790M) on exon 20 is the most frequently acquired mutation leading to resistance to tyrosine kinase inhibitors. Whether the T790M mutation occurred after tyrosine kinase inhibitor therapy or it already existed before therapy is unclear. *Methods*: Here, we developed mice with tetracycline-inducible lung-specific expression of the full-length genomic DNA of the human epidermal growth factor receptor containing an L858R mutation or both L858R and T790M mutations and evaluated de novo T790M mutation in untreated transgenic mice carrying a single L858R EGFR mutation. *Results*: The L858R mutation-associated lung adenocarcinoma acquired de novo T790 mutation without previous therapy. *Conclusions*: The results of this study suggest that lung tumors may spontaneously acquire T790M mutations without any drug-related selective pressure.

## 1. Introduction

The most common cause of cancer incidence and mortality worldwide is lung cancer. According to the World Health Organization International Agency for Research on Cancer, there were more than 2 million new cases of lung cancer and 1.7 million deaths in 2018 around the world [1]. Lung cancer is categorized into small cell lung cancer (~20%) and non-small cell lung cancer (~80%) of which adenocarcinoma is the most common [2,3,4]. Somatic mutations in the epidermal growth factor receptor (EGFR) leading to aberrant autophosphorylation of its cytosolic domain tyrosine residues and subsequent activation of survival and proliferation signal pathways are observed in lung adenocarcinoma [5]. The EGFR mutations occur in exons 18–21, which partially encodes the tyrosine kinase domain of the EGFR protein [6]. In-frame deletion of two to nine residues in exon 19 that delete the LREA motif (residues 748–751) and a point mutation (CTG to CGG) in exon 21 that causes the substitution of arginine for leucine at position 858 (L858R) are the most prevalent (85%) EGFR activating mutations [6]. Several prospective clinical trials have demonstrated the efficacy of EGFR tyrosine kinase inhibitors (TKI) (erlotinib and gefitinib) for the treatment of advanced lung adenocarcinoma patients with EGFR activating mutations [7,8]. However, in most patients, the lung tumor becomes resistant to erlotinib or gefitinib resuming detectable growth within 6 months to 2 years [9]. The substitution of methionine for threonine at position 790 (T790M) on exon 20 of the EGFR gene is the most frequent (60%) acquired mutation associated with resistance to TKI [9]. However, it is still unclear whether the T790M mutation occurs after therapy with TKI or it already exists as a minor sub-clone before therapy. In the present study, we addressed whether the T790M mutation can evolve spontaneously without any TKI therapy in mice with lung adenocarcinoma harboring the L858R EGFR mutation.

## 2. Results

### 2.1. Generation of Transgenic Mice Carrying Lung-Specific TetO-[L858R]-hEGFR or TetO-[L858R+T790M]-hEGFR BAC Transgenes

**Figure 1:**
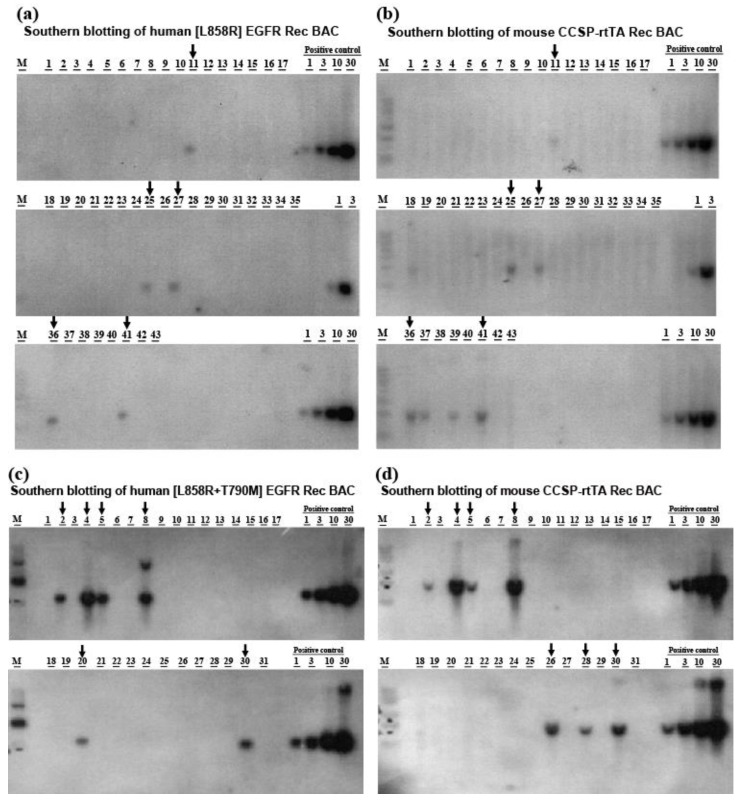
Founder mice expressing the full-length of human mutant EGFR and CCSP-rtTA. (**a**–**d**) The tail DNA from bitransgenic founder lines were analyzed by Southern blotting. The copy number of the transgenes was determined by comparing it with the control for copy number intensity.

**Figure 2:**
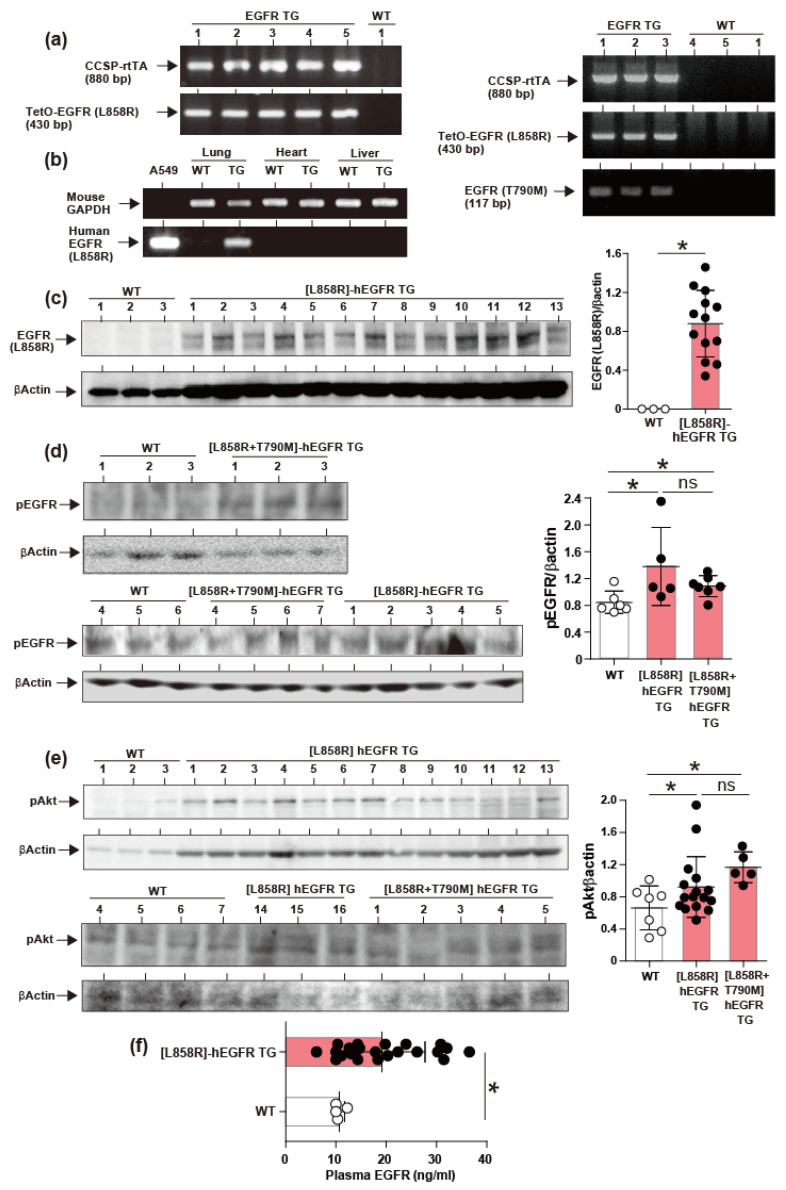
Lung-specific activation of mutant EGFR and phosphorylation of intracellular signal pathways. (**a**,**b**) Genotyping of bitransgenic mice and lung-specific expression of mutant EGFR. The uncropped Western blots have been shown in the Figure S4. (**c**–**e**) Increased expression of the [L858R]-hEGFR protein in [L858R]-hEGFR transgenic mice, phosphorylation of EGFR, and phosphorylation of Akt kinase in both [L858R]-hEGFR and [L858R+T790M] transgenic mice. (**f**) Elevated plasma level of soluble EGFR (**f**) in [L858R]-hEGFR transgenic mice. Data are expressed as the mean ± S.D. Statistical analysis by unpaired Student *t*-test. * *p* < 0.05 compared to wild type (WT) mice. hEGFR, human epidermal growth factor receptor.

Tetracycline-inducible lung-specific [L858R]-hEGFR or [L858R+T790M]-hEGFR BAC transgenic mice were generated by pronuclear injection of the CCSP-rtTA BAC expression construct together with the [L858R]-hEGFR BAC expression construct or with the [L858R+T790M]-hEGFR BAC expression construct into C57BL/6J mouse embryos (CLEA Japan Inc., Tokyo, Japan). Bitransgenic founders and germline transmission of the BAC transgenic constructs were assessed by Southern blotting using as a probe a fragment of the purified hEGFR DNA labeled with [^32^P]. Among 300 fertilized embryos that were microinjected with the [L858R]-hEGFR and CCSP-rtTA BAC expression constructs, there 43 progeny of which five contained both the [L858R]-hEGFR and the CCSP-rtTA BAC transgenes (Figure 1a,b). On the other hand, among 600 fertilized embryos that were microinjected with the [L858R+T790M]-hEGFR and CCSP-rtTA BAC expression constructs and subsequently implanted in mice, there was 105 progeny of which five contained both the [L858R+T790M]-hEGFR and the CCSP-rtTA BAC transgenes, as detected by Southern blotting (Figure 1c,d). Genotyping using tail DNA also disclosed clear bands for each hEGFR transgene and CCSP-rtTA (Figure 2a).

### 2.2. Induction of hEGFR Transgene Expression

To evaluate whether the bitransgenic (TG) mice express the transgenes specifically in the lungs they were subjected to a diet with baits containing 0.3% doxycycline and mRNA expression was evaluated by PCR. The results showed that [L858R]-hEGFR mice express the transgene specifically in the lungs (Figure 2b). The expression of the transgene proteins was significantly expressed in the lung tissues from [L858R]-hEGFR TG mice (*n* = 13) compared to WT control (*n* = 3) as evaluated by Western blotting (Figure 2c). Phosphorylation of hEGFR was significantly increased in both the [L858R]-hEGFR (*n* = 5) and [L858R + T790M]-hEGFR TG (*n* = 7) mice (Figure 2d). Aberrant activation of mutant EGFR leads to the activation of kinase pathways that control cell survival such as Akt. The phosphorylation of Akt was significantly increased in both the [L858R]-hEGFR (*n* = 16) and [L858R+T790M]-hEGFR TG (*n* = 5) mice compared to WT mice (*n* = 7) (Figure 2e). The plasma concentration of soluble EGFR was significantly increased in TG mice compared to WT mice (Figure 2F). There was no significant difference between [L858R]-hEGFR and [L858R+T790M]-hEGFR TG (*n* = 5) mice.

### 2.3. Induction of Lung Tumors in TG Mice on Doxycycline

**Figure 3:**
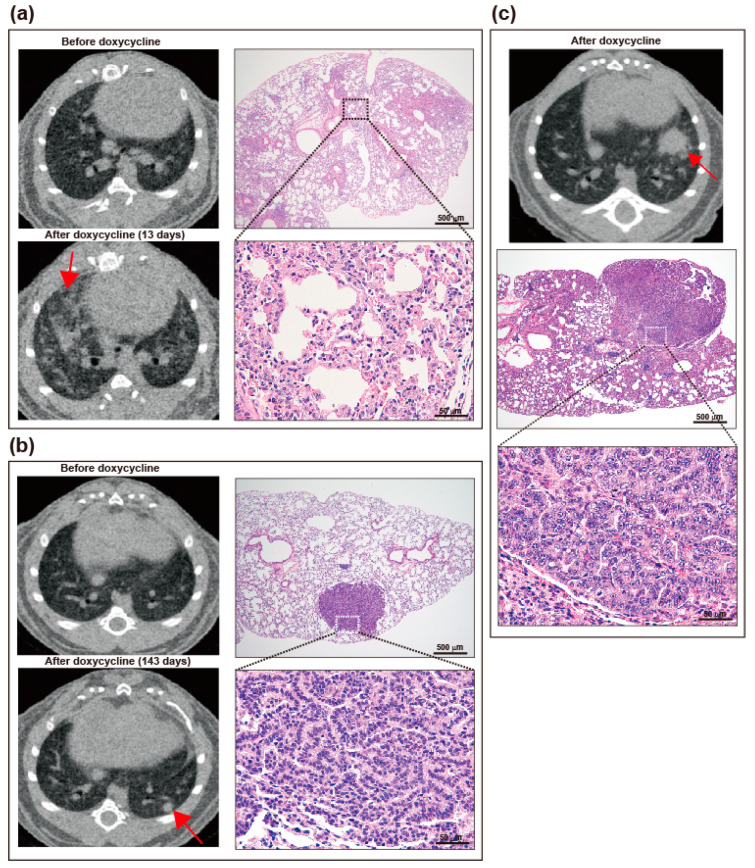
Induction of lung tumors in bitransgenic mice after doxycycline administration. CT and pathological findings of lepidic (**a**) and papillary (**b**) types of lung adenocarcinoma induced on doxycycline diet in bitransgenic mice with [L858R]-hEGFR mutation. (**c**) CT and pathological findings in a papillary type of lung adenocarcinoma induced on a doxycycline diet in bitransgenic mice with [L858R+T790M]-hEGFR mutation. Scale bars indicate 500 µm (upper panel in **a**–**c**) and 50 µm (lower panel in **a**–**c**).

Bitransgenic mice develop lung tumors in variable intervals after doxycycline administration. We screened the presence of abnormal shadows by microCT and sacrificed when the lung mass further enlarges overtime. The histopathologic types most frequently observed in the [L858R]-hEGFR bitransgenic mice were the lepidic and papillary types of adenocarcinoma according to the World Health Organization new classification of lung tumors [10]. The lepidic type of adenocarcinoma at early stages shows conserved lung architecture with thickened alveolar walls lined by carcinomatous cells and, in advanced stages, the mass shows multifocal invasive cancer cells diffusely distributed in the lung parenchyma (Figure 3a). The papillary type of adenocarcinoma appears as small peripheral nodules on CT scan and shows a focalized growth displacing surrounding normal lung parenchyma tissue (Figure 3b). The most frequent histopathologic type in the [L858R+T790M]-hEGFR bitransgenic mice was the papillary type of adenocarcinoma but, unlike that observed in the [L858R]-hEGFR bitransgenic mice, the tumor cells were more invasive and associated with increased infiltration of lymphocytes (Figure 3c).

### 2.4. Acquisition of De Novo T790M Mutant in [L858R]-hEGFR Bitransgenic Mouse Lung Tumor

**Figure 4:**
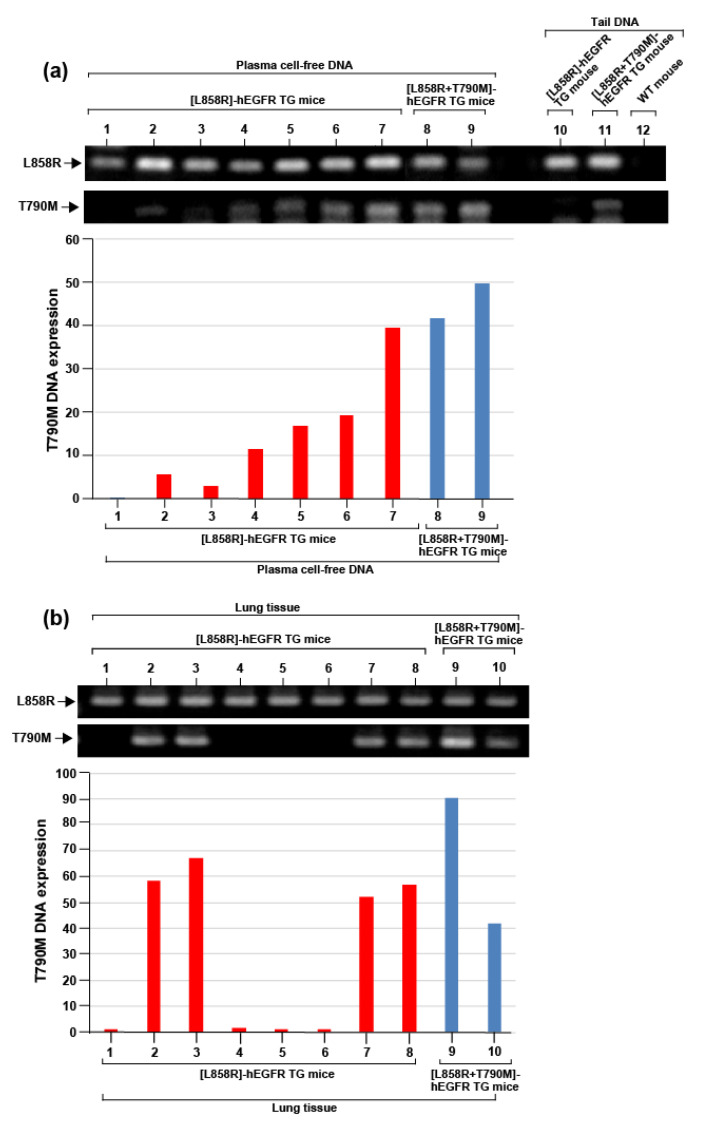
Acquired T790M mutant in [L858R]-hEGFR bitransgenic mouse lung tumor. PCR performed using plasma cell-free DNA (**a**) and lung tissue DNA (**b**) from [L858R]-hEGFR and [L858R+T790M]-hEGFR bitransgenic mice after several weeks of doxycycline administration.

Blood was drawn from seven [L858R]-hEGFR bitransgenic mice and lung tumor tissue samples were drawn from eight [L858R]-hEGFR bitransgenic mice after being on doxycycline for 12 weeks. DNA was isolated from plasma and lung tissue, and the presence of T790 mutation was evaluated by PCR. Samples taken from [L858R+T790M]-hEGFR bitransgenic mice were used for control. In addition to the L858R mutation, T790M mutation was detected in cell-free DNA from five [L858R]-hEGFR bitransgenic mice (Figure 4a). The presence of the T790M was also detected in the lung tissue DNA from four [L858R]-hEGFR bitransgenic mice (Figure 4b). None of the [L858R]-hEGFR transgenic mice showed amplification of the T790M mutant in tail DNA (Figure 4b and Figure S3).

## 3. Discussion

Here, we reported two novel murine models of lung cancer induced by lung-specific expression of the full-length genomic DNA of hEGFR containing an L858R mutation in exon 21 or a double mutation, L858R in exon 21, and T790M mutation in exon 20, and that de novo T790M mutations occur in lung tumors carrying [L858R]-hEGFR mutation.

Previous studies reported the development of lung cancer in a mouse model with a lung-specific expression of the hEGFR transgenes containing a single L858R mutation or double L858R+T790M mutation [11,12,13]. In all of these studies, the EGFR transgenic constructs were generated by cloning the hEGFR cDNA that encodes the mutant transgenes, which therefore lacked the genetic information from the non-coding regions of the hEGFR gene. An increasing amount of evidence suggests that the gene non-coding regions are involved in predisposition, progression, invasiveness, metastasis, and drug resistance of malignant tumors and that their activity correlates with patient survival [14,15,16,17,18]. To develop lung cancer mouse models that closest resemble the human disease, here we generated two transgenic mice each of them carrying a mutant transgene that contains the full-length of genomic hEGFR, and thereby the mutant transgenes have the complete coding and non-coding genetic information of the hEGFR gene. Because transcription of the mutant EGFR transgenes was tetracycline-inducible and regulated by rtTA expressed downstream of the CCSP promoter both the [L858R]-hEGFR and the [L858R+T790M]-hEGFR bitransgenic mice developed lung-specific adenocarcinomas after several weeks of oral doxycycline administration. The bitransgenic mice showed increased expression of EGFR in the lungs associated with increased phosphorylation of the Akt survival signal pathway and elevated concentration of soluble EGFR in peripheral blood.

The L858R point mutation of EGFR is one of the most frequent oncogenic drivers in lung cancer [6]. L858R-associated lung adenocarcinomas are commonly responsive to treatment with the first-generation EGFR-TKIs erlotinib and gefitinib although they inevitably become unresponsive to the therapy after a progression-free period of 6 to 24 months [7,9]. In approximately half of the patients with acquired resistance, the appearance of the secondary T790M mutation is the cause of drug resistance [9]. A question that was difficult to address so far was whether the T790M mutation already existed in the tumor or appeared de novo after TKI administration due to drug-related selective pressure. Recent in vitro studies suggest that in most cases the point mutation T790M is acquired after TKI therapy [19,20]. However, there is no evidence showing that acquired T790M mutation occurs in vivo. In our present investigation, we separated DNA from lung tissues and isolated cell-free DNA from peripheral blood of [L858R]-hEGFR bitransgenic mice under the doxycycline diet but not receiving TKI therapy, evaluated T790 mutation by PCR, and found that several [L858R]-hEGFR mice were positive for the secondary mutation. This observation suggests that de novo T790M mutation can spontaneously appear without previous TKI therapy. As the T790 mutation has been frequently associated with more advanced clinical stages of NSCLC, it is rational to speculate that increased genomic instability is the cause of this drug-resistance driving mutation [21]. The mechanism by which the T790M mutation causes resistance to TKI is also under debate. Threonine 790 is at the entrance of an ATP binding cleft. The substitution of threonine with a bulky methionine is believed to cause resistance by sterically interfering with the binding of TKIs (gefitinib, erlotinib) [22,23]. However, a recent study showed that the T790M mutation causes drug resistance by enhancing the ATP affinity of the oncogenic L858R mutant [24]. Mutation-related changes in the stability of ligand-independent dimerization of the EGFR receptor may also play role in drug resistance [25]. Overall, this study showed that mice carrying inducible lung-specific L858R-hEGFR or [L858R+T790M]-hEGFR transgene containing the full-length of genomic EGFR developed lung adenocarcinomas and that L858R-hEGFR-associated lung adenocarcinomas acquired de novo T790 mutation without previous TKI therapy.

## 4. Materials and Methods

### 4.1. Preparation of [L858R] Human EGFR and [L858R+T790M] Human EGFR Recombinant Bacterial Artificial Chromosome Expression Constructs

RP11-815K24 (196.4 kb), a human (h) EGFR bacterial artificial chromosome (BAC) clone, was selected from the human BAC Library by BLAST search of the GenBank database at NCBI. The sequence analysis of the BAC end showed that the BAC clone contains the whole 188 kb of human EGFR genomic sequence encoded by 28 exons and, additionally, a 4 kb of the 5′-flanking and 4 kb of the 3′-flanking genomic DNA. The RP11-815K24 BAC clone was obtained from the BACPAC Resources Center at the Children’s Hospital Oakland Research Institute (CHORI, Oakland, CA, USA). A tetracycline-response operon promoter element (*TetO*) was used to regulate the expression of hEGFR mutants under the control of the reverse tetracycline transactivator (rtTA), inserted downstream of the Clara cell secretory protein promoter. Recombinant *TetO*-hEGFR mutant BAC transgene expression constructs that contain the full-length coding exons and the intervening introns of hEGFR mutants were prepared by BAC recombination *genetic* engineering using *Escherichia coli*. A recombinant *TetO*-[L858R] hEGFR mutant BAC transgene expression construct containing the L858R mutation in exon 21, and a recombinant *TetO*-[L858R+T790M]-hEGFR mutant BAC transgene expression construct containing the L858R mutation in exon 21 and the T790M mutation in exon 20 were prepared (Figure S1a,b). The Red/ET Counter Selection BAC Modification Kit (Gene Bridges, Heidelberg, Germany) was used to transfer the *TetO* gene from the DNA clone to the BAC clones containing the [L858R]-hEGFR mutant or the [L858R+T790M]-hEGFR mutant.

For the construction of the *TetO*-[L858R]-hEGFR recombinant BAC clone, first, an Rpsl-kan counter-selection cassette flanked by sequences of exon 21 of the hEGFR gene was amplified by PCR. The amplified Rpsl-kan counter-selection cassette was inserted into the *TetO*-hEGFR BAC clone by Red/ET recombination. To sub-clone, the L858R mutant gene into a plasmid vector, a DNA fragment containing sequences of the 5′-untranslated region (5′-CCGCAGCATGTCAAGATCACAGATTTTGGG-3′) and 5′-flanking region (5′-CCCAAAATCTGTGATCTTGACAT-3′) of the [L858R]-hEGFR gene, an L858R mutant sequence, and sequences of the 3′ flanking region (5′-TCTTCCGCACCCAGCAGTTTGGC-3′), and the 3′-untranslated region (5′-GCCAAACTGCTGGGTGCGGAAGAGAAAGAA-3′) of the hEGFR gene exon 21 were amplified by PCR. The PCR DNA fragment was inserted into a pGEM-T easy plasmid vector by Red/ET recombination in transformed competent cells (*E. coli*). Ampicillin-resistant colonies were picked up and construct clones containing the L858R mutant were screened using the colony PCR method. The Rpsl-kan counter-selection cassette inserted in exon 21 of the *TetO*-hEGFR BAC clone was then replaced by the DNA fragment containing the L858R mutant by Red/ET recombination in *E. coli* (Figure S1a). The presence of the L858R mutant in the *TetO*-[L858R]-hEGFR recombinant BAC clone was confirmed by sequence analysis (Figure S1c).

For the construction of the *TetO*-[L858R+T790M]-hEGFR recombinant BAC clone, first, a Rpsl-kan counter-selection cassette flanked by sequences of exon 20 of the hEGFR gene was amplified by PCR. The amplified Rpsl-kan counter-selection cassette was inserted by Red/ET recombination into a *TetO*-[L858R]-hEGFR BAC clone prepared in advance. A plasmid vector (Genewiz, Saitama, Japan) that has a DNA fragment containing a sequence of the 5′-flanking region (5′-GTCCATGTGCCCCTCCTTCTGGCCACCATG-3′) of the [T790]-hEGFR gene, a T790M mutant sequence, and a sequence of the 3′-flanking region (5′-ACGGGGAGGGGAGATAAGGAGCCAGGATCC-3′) of the [T790]-hEGFR gene was used for sub-cloning the T790M mutant DNA fragment. After transformation in competent cells, single colonies were picked up, and then the plasmids were extracted and treated with restriction enzymes to separate the DNA fragment containing the T790M mutant fragment. The Rpsl-kan counter-selection cassette inserted in exon 20 of the *TetO*-[L858R]-hEGFR BAC clone was replaced by a DNA fragment containing the T790M mutant sequence by Red/ET recombination in *E. coli* (Figure S1b). The presence of the T790M mutant in the *TetO*-[L858R+T790M]-hEGFR recombinant BAC clone was confirmed by sequence analysis (Figure S1d).

The *TetO*-[L858R]-hEGFR and the *TetO*-[L858R+T790M]-hEGFR BAC transgenic constructs were purified for microinjection [26]. Briefly, the BAC transgenic constructs were extracted from the *E. coli* culture using the Nucleobond Plasmid Purification kit (MACHEREY-NAGEL, Düren, Germany), and then linearized overnight with PI-SceI endonuclease (New England Biolabs, Ipswich, MA, USA). The linearized BAC DNAs were separated by pulsed-field gel electrophoresis and extracted from the gel by electroelution. After dialysis against TE (Tris and ethylenediaminetetraacetic acid) buffer, aliquots of the BAC DNA constructs were subjected to pulsed-field gel electrophoresis for size and quality control and then stored at 4 °C until microinjection.

### 4.2. Preparation of the CCSP-rtTA Recombinant BAC Expression Construct

A mouse Clara cell secretory protein (CCSP) BAC clone, RP23-223J21 (170.1 bp), was selected from the C57BL/6J Mouse BAC Library by searching the mouse BAC ends database at National Center for Biotechnology Information (NCBI). The BAC end sequences indicated that the BAC clone contains the whole 4 kb of mouse CCSP genomic sequence, with an additional 132 kb and 34 kb of the 5′-, and 3′-flanking genomic DNAs, respectively. We used this CCSP BAC clone to prepare the rtTA expression vector. The BAC clone was obtained from the BACPAC Resources Center at Children’s Hospital Oakland Research Institute (CHORI, Oakland, CA, USA) (Figure S2a).

For the construction of the CCSP-rtTA recombinant BAC clone, first, an Rpsl-kan counter-selection cassette was inserted in the translation initiation codon of the mouse CCSP gene exon 1. For this purpose, a DNA fragment containing a sequence of the 5′-flanking region of the mouse CCSP gene translation initiation codon (5′-CTACAGACACCAAAGCCTCCAACCTCTACC-3′), the Rpsl-kan cassette, and a sequence of the 3′-flanking region of the mouse CCSP gene translation initiation codon (5′-AAGATCGCCATCACAATCACTGTGGTCATG-3’) was amplified by PCR (CCSP Rpsl-kan cassette break-in DNA fragment). The amplified Rpsl-kan counter-selection cassette was inserted by Red/ET recombination in the mouse CCSP BAC clone (mCCSP-Rpsl-kan-BAC clone). To insert the rtTA gene DNA sequence into the mCCSP-Rpsl-kan-BAC clone, we prepared a rtTA-SV40 polyA fragment containing sequences of the flanking regions where the Rpsl-kan cassette was inserted (rtTA-SV40 polyA repair fragment). The Rpsl-kan counter-selection cassette inserted in the translation initiation codon of the mouse CCSP gene exon 1 was replaced by the rtTA-SV40 polyA repair fragment by Red/ET recombination in *E. coli* (Figure S2a). The presence of the rtTA-SV40 polyA fragment in the CCSP-rtTA recombinant BAC clone was confirmed by sequence analysis (Figure S2b). The CCSP-rtTA recombinant BAC clone was then purified, linearized, and prepared for microinjection as described above.

### 4.3. Ethical Statement

The Recombinant DNA Experiment Safety Committee (Approval No: I-669; Date: 2017/01/14; Approval No I-628; Date 2013/09/19) and the Committee on Animal Investigation of Mie University (Approval No: 29–23; Date: 2018/01/15; Approval No 2019-5; Date 2019/07/22) approved the protocols of the study. All animal procedures were performed under the institutional guidelines of Mie University and following the internationally approved principles of laboratory animal care published by the National Institute of Health (https://olaw.nih.gov/).

### 4.4. Screening of Lung Tumors by Micro-Computed Tomography (CT)

Micro-CT (Latheta LCT-200, Hitachi Aloka Medical, Tokyo, Japan) was used to screen the presence of lung tumors in the mouse models. Acquisition of data was performed with mice in prone position under isoflurane inhalation anesthesia with respiratory gating as previously described [27,28,29].

### 4.5. Plasma Sampling

After mouse euthanasia, blood was sampled from the jugular vein and collected in heparinized tubes. The blood samples were then stored on ice before centrifuging at 10,000 rpm for 3 min at 4 °C. Plasma samples were collected in 1.5 mL tubes and stored at −80 °C until analysis. For the separation of cell-free DNA (cfDNA) from plasma, the QIA Amp DNA blood mini kit from QIAGEN (Valencia, CA, USA) was used.

### 4.6. Lung Tissue Examination

The lungs and other organs were removed after euthanasia by an overdose of anesthesia after flushing the pulmonary circulation with saline. The lungs were perfused with 10% neutral buffered formalin, fixed in formalin overnight and embedded in paraffin. Lung tissue sections of 5 μm width were prepared, deparaffinized, and then washed many times with phosphate-buffered saline. The tissue sections were then prepared for hematoxylin/eosin staining and examination of the lungs was performed using an Olympus BX50 microscope with a plan objective combined with an Olympus DP70 digital camera (Tokyo, Japan).

### 4.7. Biochemical Analysis

The plasma concentration of soluble EGFR was measured using a commercially available enzyme immunoassay kit (EGFR Duo Set ELISA, R&D Systems, Minneapolis, MN, USA) following the manufacturer’s instructions.

### 4.8. Western Blotting

The lung tissues of mice treated with doxycycline were homogenized in radioimmunoprecipitation assay (RIPA) buffer supplemented with inhibitors of protease/phosphatase. After centrifugation, the protein content was measured using the Pierce BCA protein assay kit (Thermo Fisher Scientific Incorporation, Waltham, MA, USA). Samples with an equal amount of protein were separated by sodium dodecyl sulfate-polyacrylamide gel electrophoresis and then Western blotting was performed using anti-phospho-Akt, anti-βactin, and anti-pEGFR antibodies (Cell Signaling, Danvers, MA, USA) as previously described 12. The intensity of the bands was quantified by densitometry using the public domain NIH ImageJ program (wayne@codon.nih.gov; Wayne Rasband, NIH, Research Service Branch, Bethesda, MD, USA).

### 4.9. Evaluation of Gene Expression by PCR

The total RNA was extracted from mouse organs by Trizol Reagent (Invitrogen, Carlsbad, CA, USA) and cDNA was synthesized using 2 µg of total RNA, oligo-dT primers, and reverse transcriptase (Toyobo Life Science Department, Osaka, Japan). Quantitative real-time RT-PCR was carried out using the Applied Biosystem 7500 Real-Time PCR System. The data were then analyzed using the 7500 software from Applied Biosystems and the expression of each gene was normalized by the transcription level of glyceraldehyde-3-phosphate dehydrogenase (GAPDH). In some experiments, the PCR products were run on a 2% agarose gel, and the bands were visualized by ethidium bromide staining and ultraviolet trans-illumination. The specific primer sequences were as follows; a forward primer for WT EGFR exon 21, CATGAACTACTTGGAGGACC, reverse primer for WT EGFR exon 21, CACCCAGCAGTTTGGCCA, reverse primer for WT EGFR exon 21, CACCCAGCAGTTTGGCCC, a forward primer for WT EGFR exon 20, CACCGTGCAGCTCATCAC, reverse primer for WT EGFR exon 20, CACCCAGCAGTTTGGCCA, reverse primer for WT EGFR exon 20, CACCCAGCAGTTTGGCCC. Primers used for CCSP-rtTA, rtTA forward primer, AGAAACAGCTAAAGTGCGAAAGC, CCSPBI reverse primer CTGGTCTAGATGGGCTCTCTCC, primers used for TetO-EGFR, forward TRE3Gpro primer GTATAAGCTTTAGGCGTGTACGG, reverse hEGFRTetOBI primer TTACCTTTCTTTTCCTCCAGAGC.

### 4.10. Statistical Analysis

All data are expressed as the mean ± standard deviations (SD). The statistical difference between two variables was calculated by the Student *t*-test or Mann–Whitney U test. Statistical analyses were carried out using the Graphpad Prism version 8.0.1 (GraphPad Software, San Diego, CA, USA). A *p* < 0.05 was considered as statistically significant.

## 5. Conclusions

In conclusion, in the present study, we showed that mice carrying inducible lung-specific L858R-hEGFR or [L858R+T790M]-hEGFR transgene containing the full-length of genomic EGFR developed lung adenocarcinomas and that L858R-hEGFR-associated lung adenocarcinomas acquired de novo T790 mutation without previous TKI therapy.

## 6. Patents

E.C.G. and C.N.D-G. have issued a patent on the bitransgenic mutant EGFR models used in the present study.

## Supplementary Materials

The following are available online at https://www.mdpi.com/2072-6694/12/10/3074/s1, Figure S1. Human epidermal growth factor receptor (EGFR) bacterial artificial chromosome (BAC) constructs. Figure S2. Mouse Clara cell secretory protein (CCSP) bacterial artificial chromosome (BAC) construct, Figure S3. Polymerase chain reaction performed using tail DNA from wild type mice, [L858R] hEGFR and L858R/T790M hEGFR transgenic mice, Figure S4. Uncropped western blot figures.
