# Supplementary material for: De Novo T790M Mutation in an L858R Epidermal Growth Factor Receptor Mutant-Associated Lung Adenocarcinoma
Source: Cancers (Basel). 2020 Oct 21;12(10):3074. doi: 10.3390/cancers12103074 (PMC7589092; doi:10.3390/cancers12103074)
Supplement: Supplementary file 1 [file cancers-12-03074-s001.pdf]

Supplementary materials: De Novo T790M Mutation in an L858R Epidermal Growth Factor Receptor Mutant-Associated Lung Adenocarcinoma

Takumi Fujiwara, Tetsu Kobayashi, Taro Yasuma, Corina N. D' Alessandro-Gabazza, Masaaki Toda, Hajime Fujimoto, Kentaro Fujiwara, Atsuro Takeshita[2], Kota Nishihama, Tomohito Okano, Valeria Fridman D'Alessandro, Yoshiyuki Takei, Osamu Hataji, Esteban C Gabazza

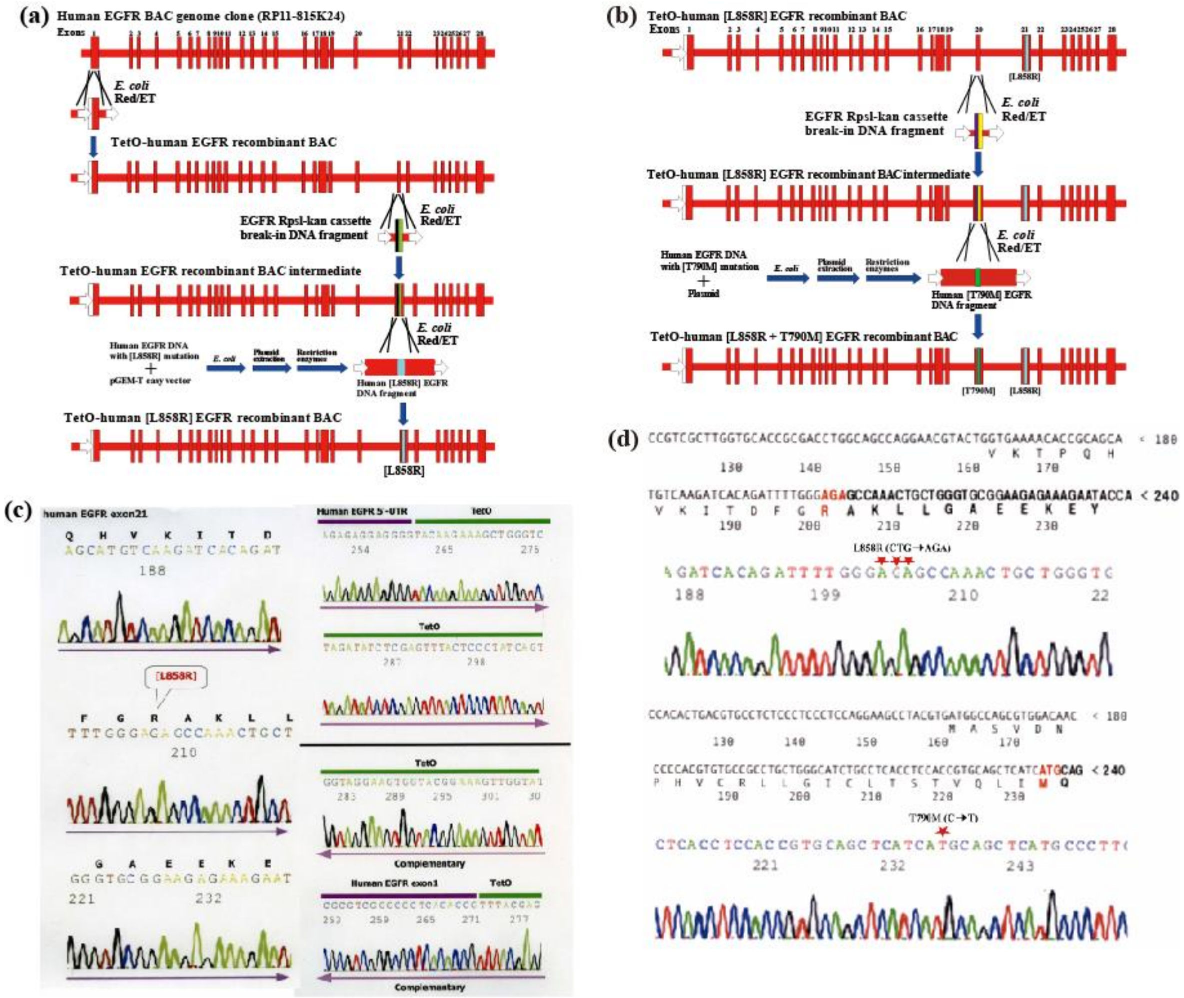

Figure S1. Human epidermal growth factor receptor (EGFR) bacterial artificial chromosome (BAC) constructs. Structure of the human EGFR BAC clone composed of the full-length coding sequence and 5′- and 3′-flanking sequences and diagrams of. the transgenes construction. The tetracycline-response operon promoter element (TetO) gene was transferred to the BAC clones containing the [L858R]-hEGFR mutant or the [L858R+T790M]-hEGFR mutant (**a, b**). Sequence analysis of the constructs to confirm the presence of [L858R] mutation or double [L858R+T790M] mutation (**c, d**).

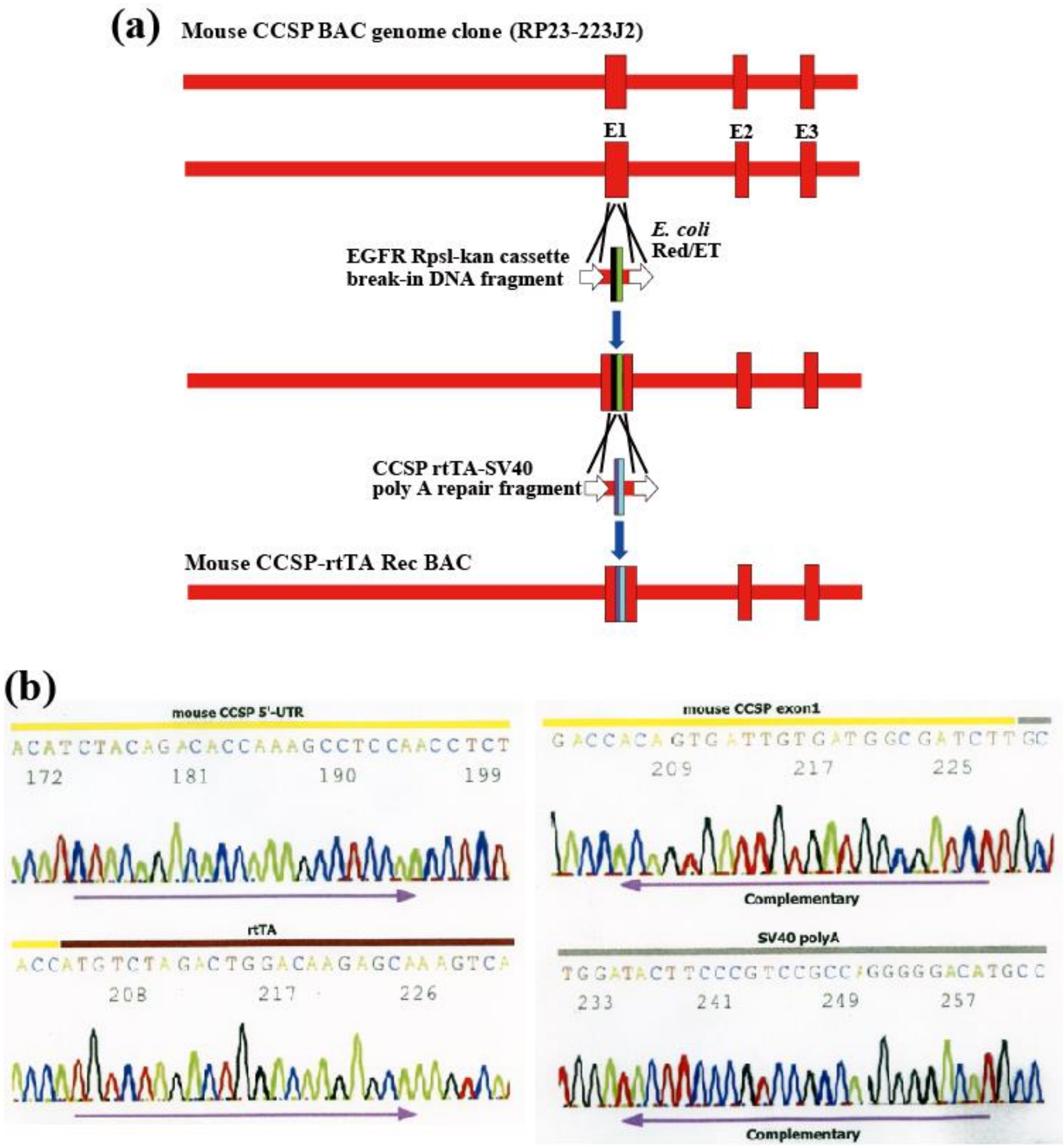

Figure S2. Mouse Clara cell secretory protein (CCSP) bacterial artificial chromosome (BAC) construct. Structure of the mouse CCSP BAC clone composed of the full-length coding sequence and 5′- and 3′-flanking sequences and diagram of the transgene construction (**a**). The reverse tetracycline transactivator (rtTA) gene was transferred to the BAC clone downstream of the CCSP promoter. Sequence analysis of the construct to confirm the presence of the rtTA sequence (**b**).

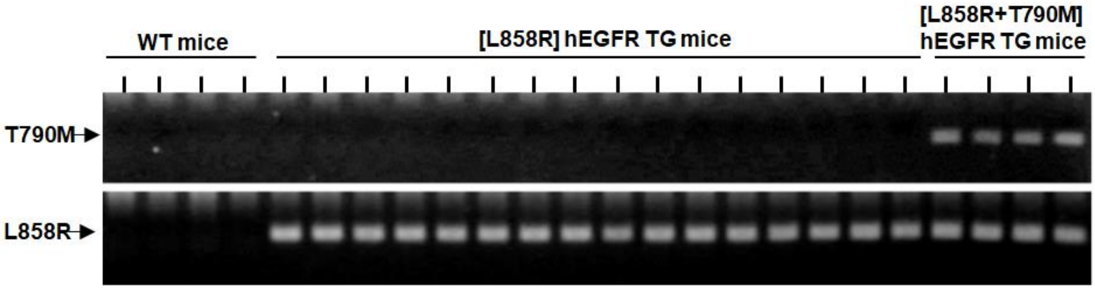

Figure S3. Polymerase chain reaction performed using tail DNA from wild type mice, [L858R] hEGFR and L858R/T790M hEGFR transgenic mice. DNA was extracted from the. tail tissue and polymerase chain reaction (PCR) was carried out using specific primers and experimental conditions as described under materials and methods. hEGFR, human epidermal growth factor receptor; WT, wild type; TG, transgenic.

Fig. 2a left

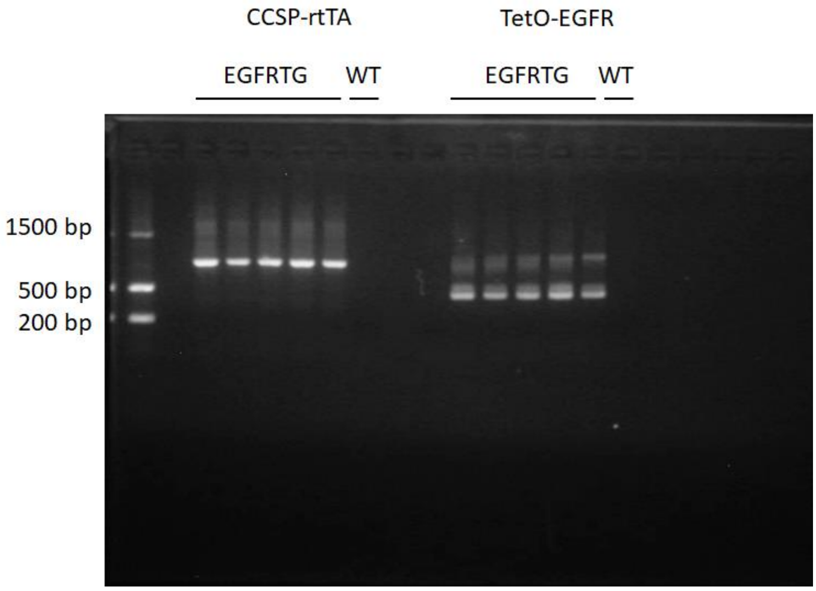

Fig. 2a right

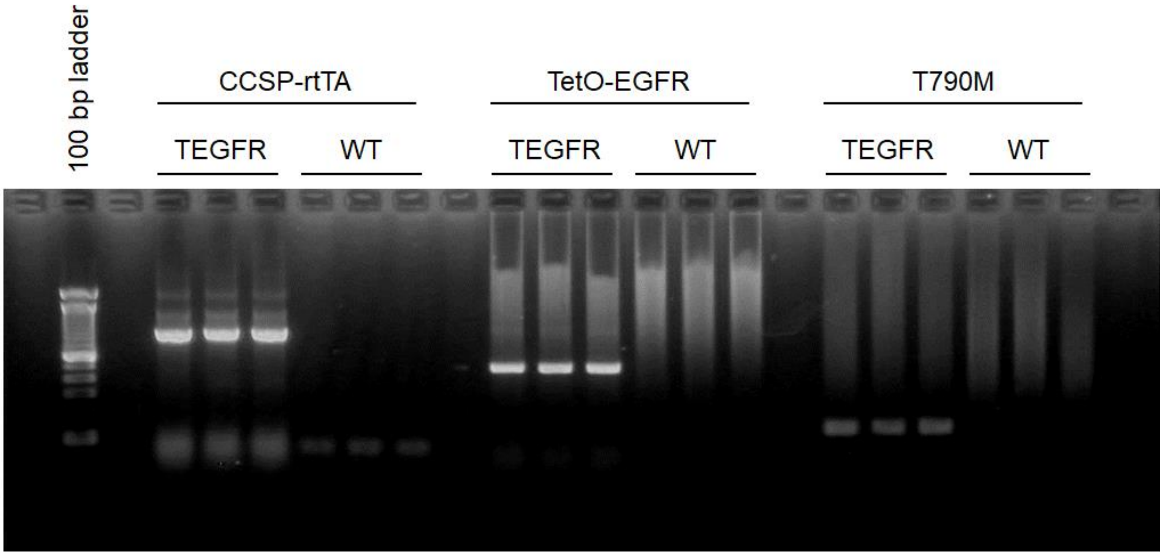

Fig. 2b

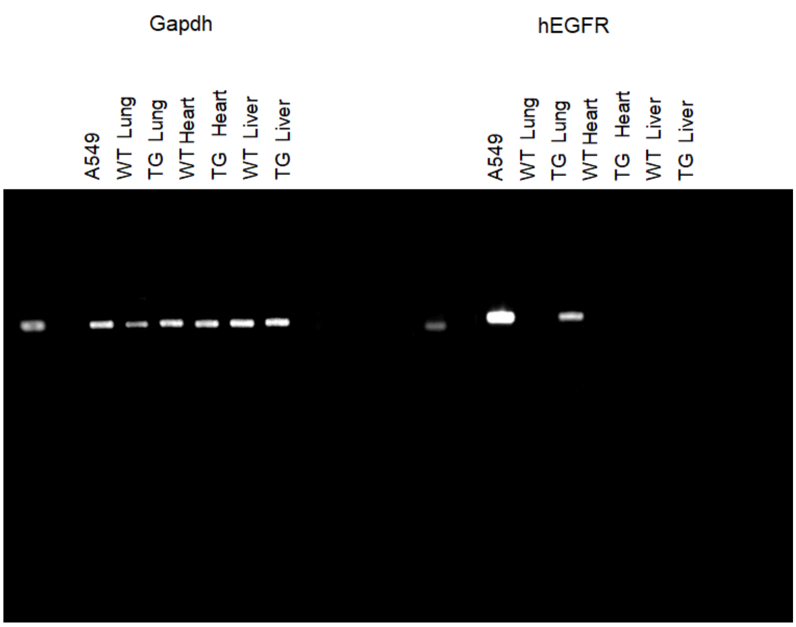

Fig. 2c

WB anti-human EGFR (L858R)

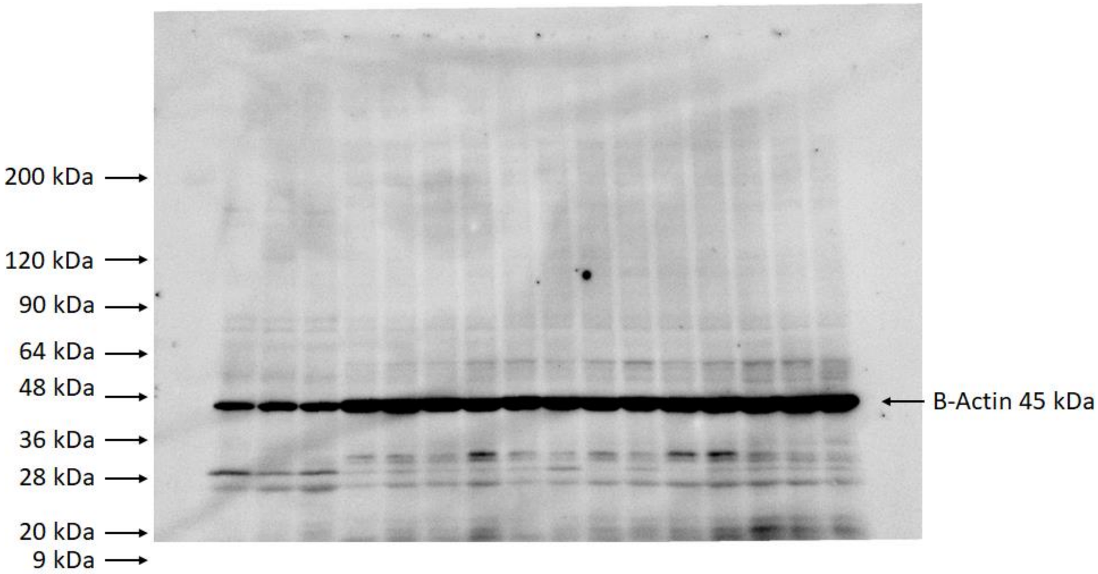

Fig. 2c

WB anti-human EGFR (L858R)

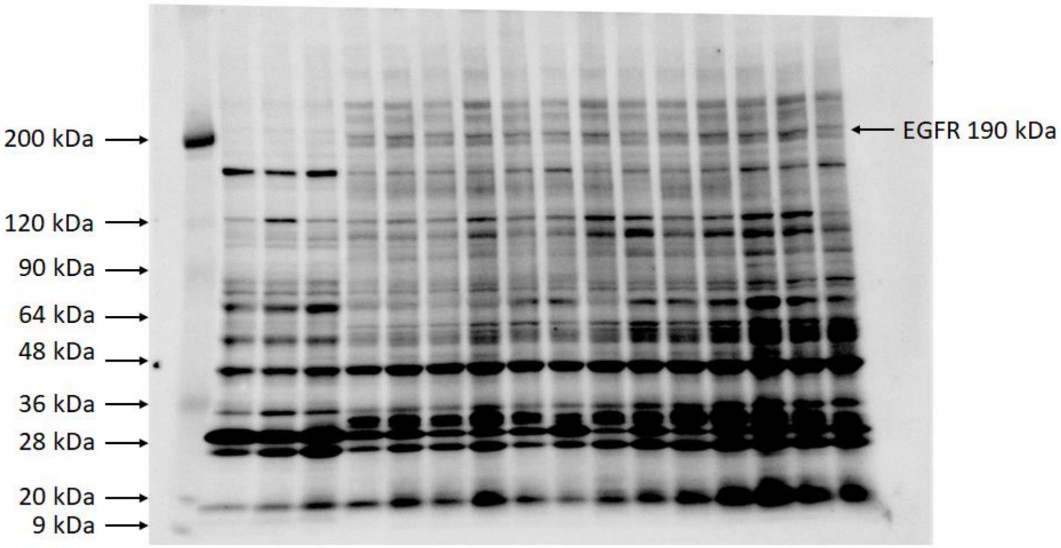

Fig. 2d

pEGFR β-Actin

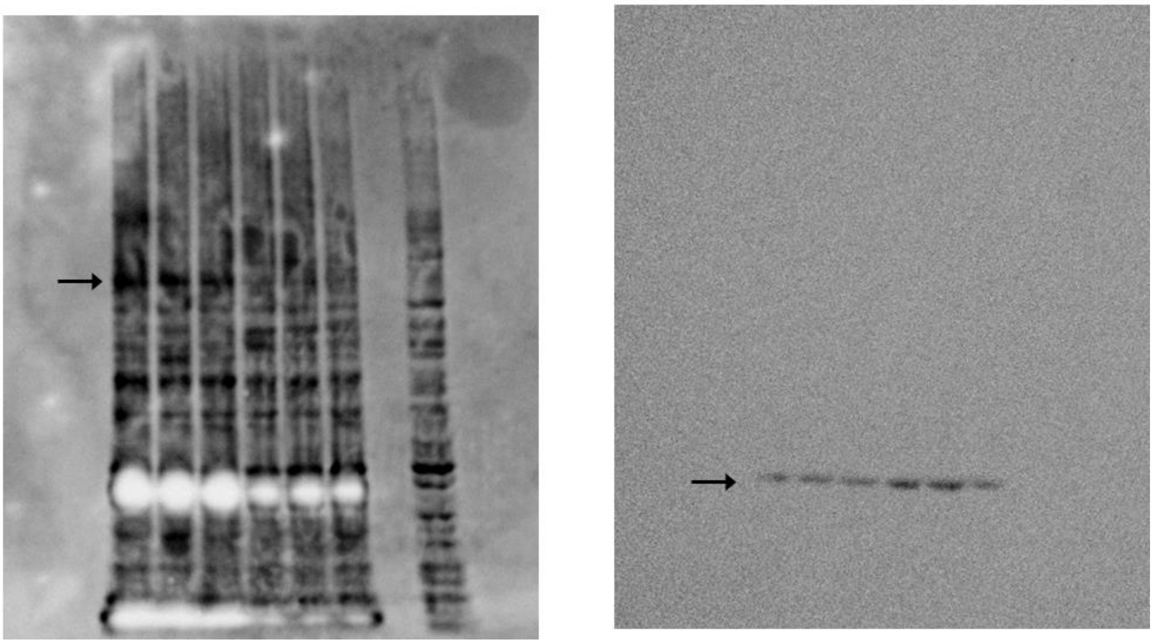

Fig. 2e

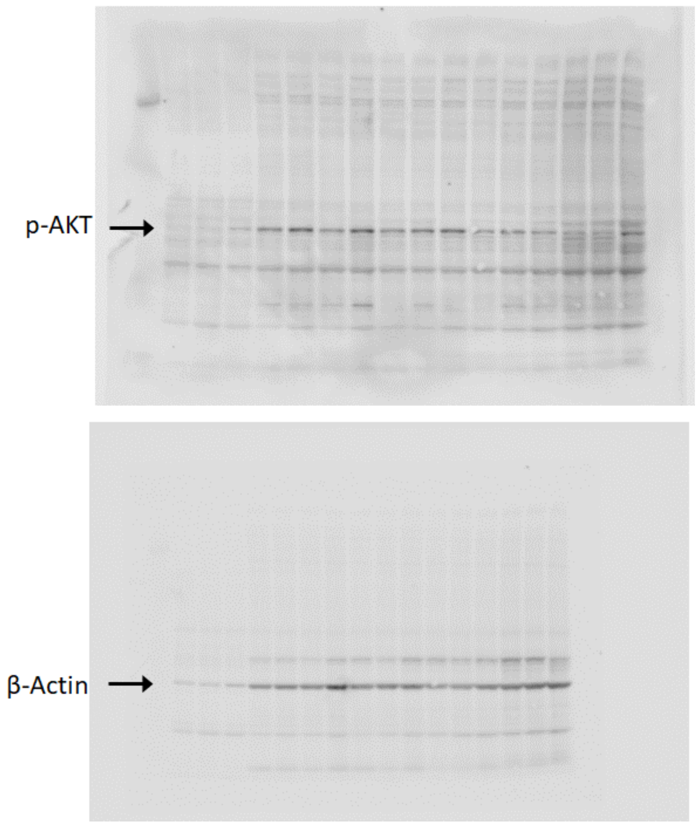

Fig. 2e

1° Ab: rabbit anti-phospho-AKT

2° Ab: goat anti-rabbit IgG-HRP

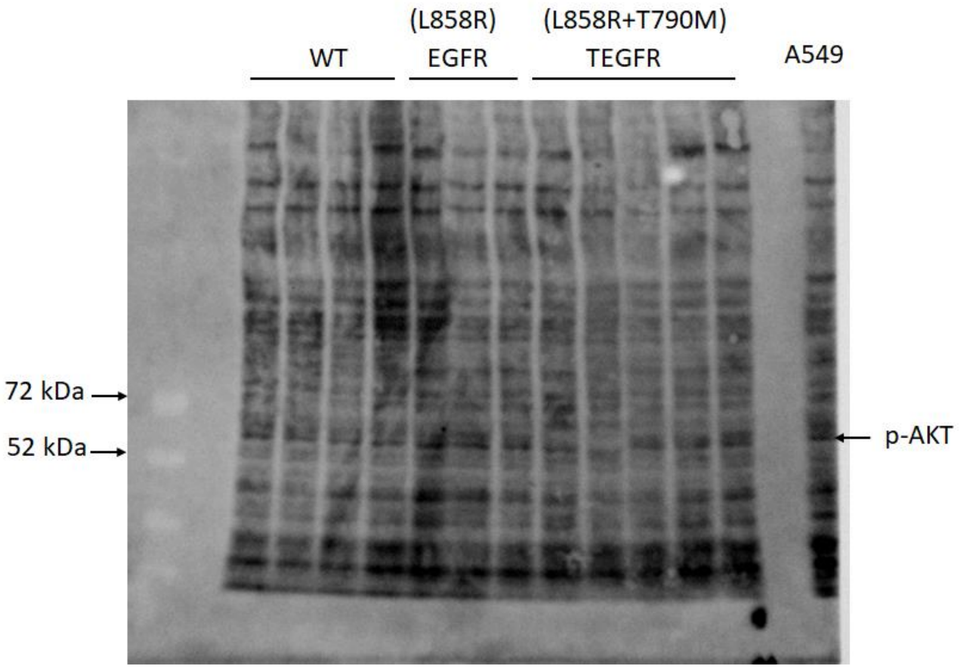

Fig. 2e

1° Ab: rabbit anti-β-actin
2° Ab: goat anti-rabbit IgG-HRP

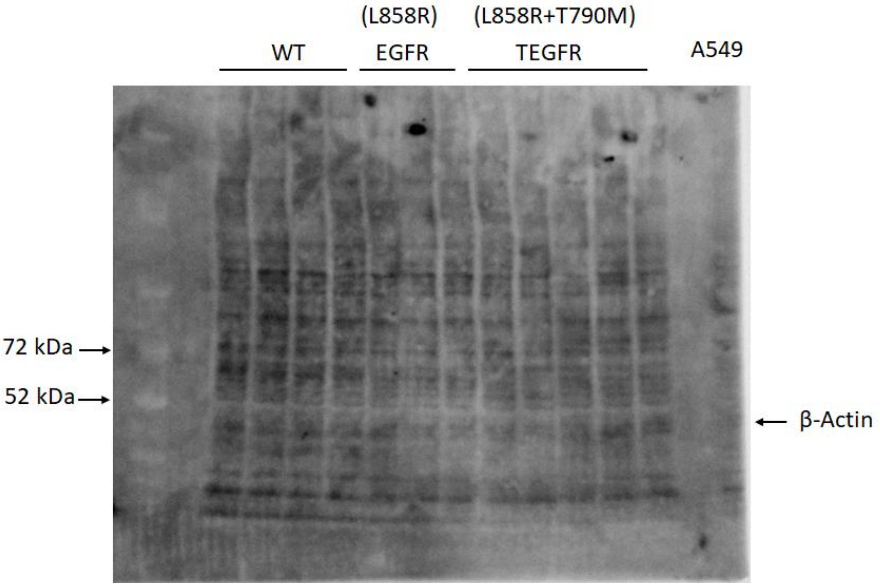

Figure S3

T790M mutation specific PCR

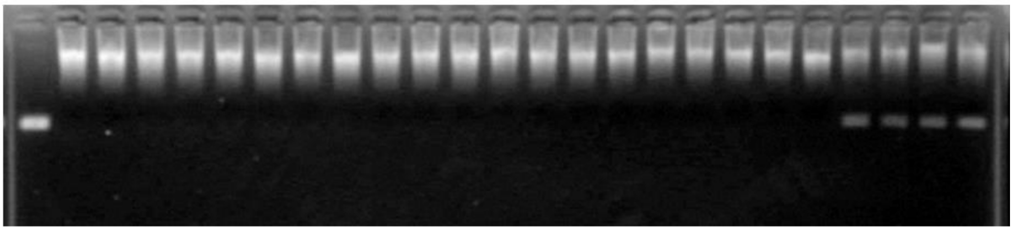

L858R mutation specific PCR

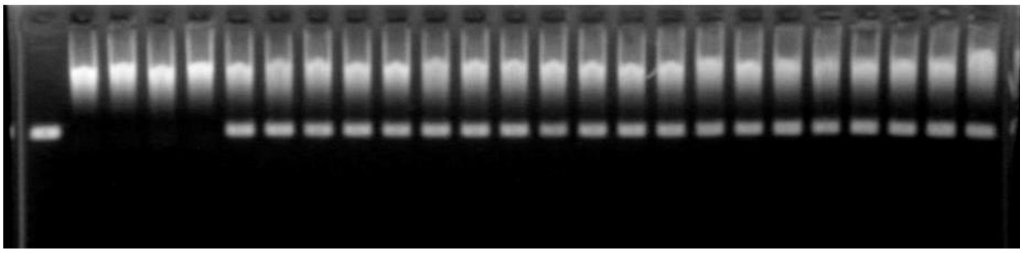

Fig. 4a

L858R

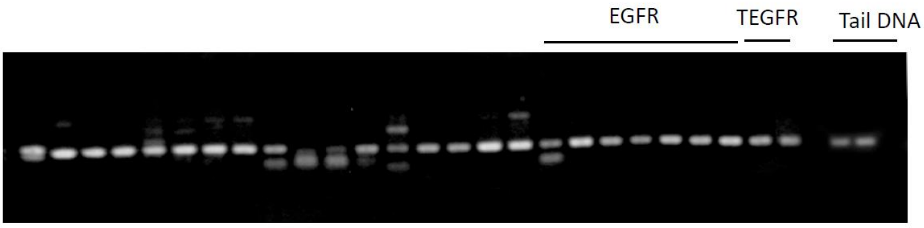

T790M

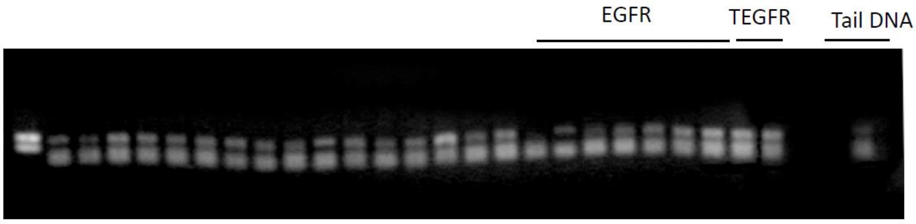

Fig. 4b

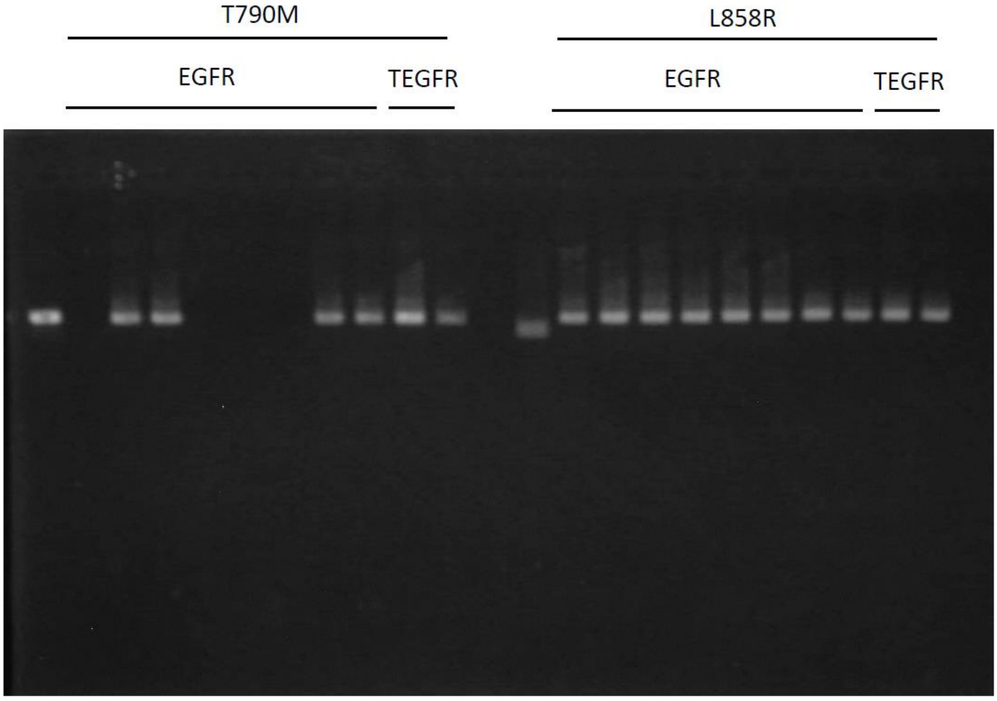

Figure S4. Uncropped western blot figures.

© 2020 by the authors. Licensee MDPI, Basel, Switzerland. This article is an open access article distributed under the terms and conditions of the Creative Commons Attribution (CC BY) license (http://creativecommons.org/licenses/by/4.0/).
